# Factors Associated with Hyperkalemia in Chronic Kidney Disease: Insights from a Cohort with Measured Glomerular Filtration Rate

**DOI:** 10.1159/ajn/ablag008

**Published:** 2026-08-28

**Authors:** Justina Motiejunaite, Martin Flamant, Alexandre Lahens, Timothée Fearon, Nahid Tabibzadeh, Anne Boutten, François Vrtovsnik, Benedicte Stengel, Natalia Alencar de Pinho, Emmanuelle Vidal-Petiot

**Affiliations:** a Service de Physiologie et Explorations Fonctionnelles, Assistance Publique - Hôpitaux de Paris, Hôpital Bichat-Claude Bernard, Paris, France; b Université Paris Cité, Paris, France; c Centre for research in Epidemiology and Population Health (CESP), Paris-Saclay University, Inserm U1018, Versailles Saint-Quentin University, Clinical Epidemiology Team, Villejuif, France; d INSERM U1148, Université Paris Cité, LVTS, Paris, France; e Institut Curie, Unité d’oncologie computationnelle, INSERM U1331, Saint Cloud, France; f Centre de Recherche des Cordeliers, INSERM, Sorbonne Université, Université Paris Cité, Laboratoire de Physiologie Rénale et Tubulopathies, Paris, France; g CNRS EMR 8228 – Unité Métabolisme et Physiologie Rénale, Paris, France; h Laboratoire de Biochimie, Assistance Publique – Hôpitaux de Paris, Hôpital Bichat-Claude Bernard, Paris, France; i Service de Nephrologie, Assistance Publique – Hôpitaux de Paris, Hôpital Bichat-Claude Bernard, Paris, France

**Keywords:** Chronic kidney disease, Hyperkalemia, Epidemiology, Measured GFR

## Abstract

**Introduction:**

Hyperkalemia is a life-threatening disorder in chronic kidney disease (CKD), yet the contribution of key factors including age, sex, body mass index, and proteinuria remain controversial. We aimed to identify factors associated with chronic hyperkalemia in a large cohort of patients with gold-standard measured glomerular filtration rate (GFR) and standardized plasma potassium measurements.

**Methods:**

This cross-sectional study of 5,046 adults with non-dialysis CKD referred for kidney workup, including radio-isotopic measured GFR, defined hyperkalemia as a plasma potassium concentration ≥5.0 mmol/L or the use of potassium binders. We analyzed demographic, clinical, pharmacological, and biochemical risk factors for hyperkalemia using logistic regression models.

**Results:**

Mean measured GFR was 59 ± 26 mL/min/1.73 m^2^, mean age was 52 ± 15 years, 42% were women, 22% were of sub-Saharan African ancestry, 25% had diabetes, and 36% were kidney transplant recipients. The prevalence of hyperkalemia was 6.4% and increased with declining measured GFR, from 1% in CKD stages 1–2–8% in CKD stage 3 and 21% in stages 4–5. In the multivariable model, measured GFR remained strongly associated with hyperkalemia (OR 1.65 [1.51–1.81] per 10 mL/min/1.73 m^2^ decrease), an effect amplified in renin-angiotensin-aldosterone system inhibitor (RAASi) users (1.82 [1.61–2.07]) (*p* for interaction 0.036). Other independently associated factors were diabetes (OR 1.67 [1.38–2.17]), RAASi and calcineurin inhibitor (OR 2.30 [1.72–3.11] and 1.39 [1.06–1.83] respectively), higher urinary albumin-to-creatinine ratio, and lower venous bicarbonate level, whereas female sex (OR 0.71 [0.55–0.92]), sub-Saharan African ancestry (HR 0.68 [0.48–0.94]), and loop diuretics (OR 0.71 [0.51–0.98]) were protective, and age and body mass index showed no independent association.

**Conclusion:**

In this large cohort, across the entire range of gold-standard measured GFR, hyperkalemia is driven primarily by reduced kidney function, diabetes, and RAASi, whereas protective factors were female sex, sub-Saharan African ancestry, and loop diuretic therapy. These robust findings, based on unbiased GFR assessment, identify key contributors for individualized prevention of hyperkalemia in CKD.

## Plain Language Summary

This study examined why some people with chronic kidney disease (CKD) develop high blood potassium levels, a condition called hyperkalemia. Hyperkalemia can cause serious heart rhythm problems and may limit the use of important kidney- and heart-protective medications. Previous studies have reported conflicting results regarding the role of factors such as age, sex, body weight, and proteinuria. We therefore analyzed data from more than 5,000 adults with non-dialysis CKD who underwent a detailed kidney evaluation, including direct measurement of kidney function using a gold-standard method. Overall, 6.4% of participants had hyperkalemia, and the frequency increased markedly as kidney function declined. Hyperkalemia affected only 1% of patients with mild CKD but more than 20% of those with advanced CKD. Reduced kidney function was the strongest risk factor. Diabetes and treatment with renin-angiotensin-aldosterone system inhibitors (RAAS inhibitors), commonly prescribed to protect the kidneys and heart, were also strongly associated with hyperkalemia. This risk was even greater in patients with more severe kidney impairment. Lower blood bicarbonate levels, higher albuminuria, and use of calcineurin inhibitors were additional risk factors. In contrast, women, individuals of sub-Saharan African ancestry, and patients treated with loop diuretics were less likely to develop hyperkalemia. Importantly, age and body mass index were not independently associated with risk after accounting for other factors. These findings improve understanding of hyperkalemia in CKD and may help clinicians identify high-risk patients and tailor prevention strategies more effectively.

## Introduction

Hyperkalemia is a frequent electrolyte disorder in patients with chronic kidney disease (CKD), yet its reported prevalence varies considerably depending on the population and the definition used [1–6]. As kidney function declines, the capacity to excrete potassium becomes progressively impaired, increasing the risk of hyperkalemia [7, 8] and contributing to a range of life-threatening adverse outcomes and complicating the use of kidney- and cardio-protective therapies [9–11] that form the cornerstone of CKD management.

Multiple factors influence the development of hyperkalemia in patients with CKD. Reduced glomerular filtration rate (GFR), disturbances in kidney tubular function, and potassium shift out of cells (often driven by metabolic acidosis) are key factors directly impairing potassium homeostasis [12]. The risk of hyperkalemia is inversely proportional to kidney function [1], the independent contribution of proteinuria being unclear [1, 13, 14]. Pharmacological treatments commonly prescribed in CKD – such as renin-angiotensin-aldosterone inhibitors (RAASis) – further elevate the risk of hyperkalemia, which in turn often leads to therapy discontinuation, compromising long-term cardiorenal benefits [15]. Diabetes and anthropometric parameters such as low body mass index (BMI) also modulate potassium balance and may contribute to increased risk of hyperkalemia [1, 2, 16, 17]. Although demographic characteristics such as age and sex are allegedly key contributors, prior studies in CKD cohorts have yielded conflicting results regarding their association with hyperkalemia [1, 2, 14, 17, 18].

Understanding the interplay among these factors is essential for risk stratification, patient monitoring, and optimizing therapy to avoid the occurrence of hyperkalemia. As GFR itself is the central contributor to hyperkalemia in CKD, much of the existing evidence is limited by reliance on estimated GFR, which may lack precision and/or display systematic bias depending on age, sex, anthropometric parameters, and comorbidities [19], and by potassium measurements, often obtained from heterogeneous clinical settings or healthcare databases. The aim of our study was to describe the factors associated with chronic outpatient hyperkalemia in a cohort of non-dialysis CKD patients across the entire range of kidney function, relying on gold-standard measured GFR and simultaneous, standardized, plasma potassium measurement.

## Methods

### Study Design and Participants

We analyzed data from 5,124 consecutive adult patients with CKD stages 1–5, who were referred to the physiology unit of Bichat Hospital (AP-HP, Paris, France) for kidney function evaluation as part of routine clinical care from September 2006 through January 2024. Pregnant women or patients on dialysis were excluded from the analysis (*n* = 14), as well as patients with missing data for measured GFR (*n* = 37) or plasma potassium (*n* = 27) (Fig. 1).

**Fig. 1.**
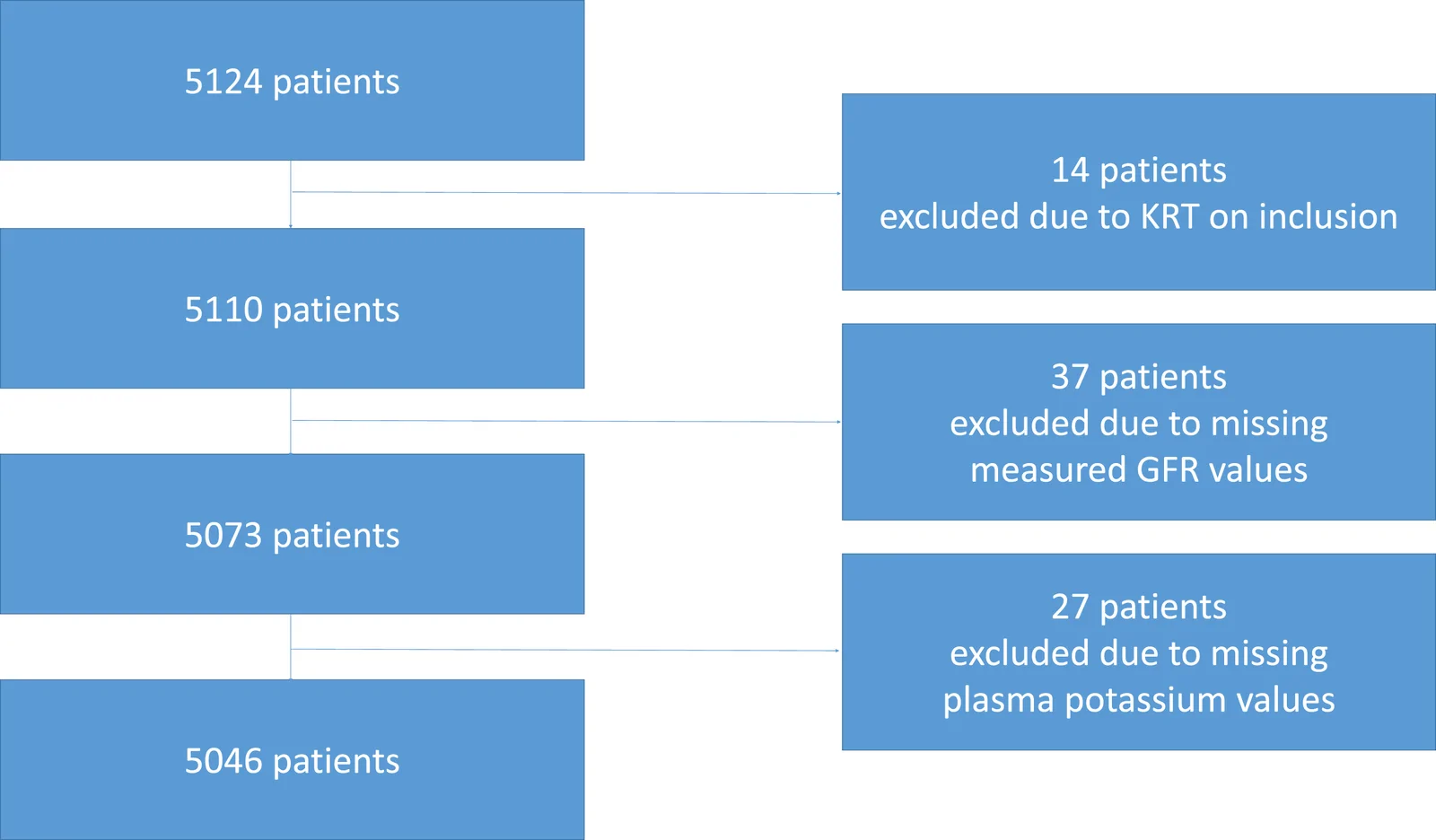
Flowchart of the study population.

All patients signed informed consent for the scientific use of anonymized data. The study was approved by the local Ethics Committee and was conducted in accordance with good clinical practices and the Declaration of Helsinki.

### Procedures

Patients were admitted for a 5-h hospital stay, starting at 08:00 am, in fasting conditions. Medical history, current treatments, anthropometric data, and a large set of clinical variables were collected by the attending nurse and physician. Blood pressure was measured in standardized conditions after at least 5-min rest and defined as controlled if the average of three measurements was <140/90 mm Hg.

Blood and urine samples were collected between 8:00 am and 09:00 am for biochemistry analyses including plasma potassium, sodium and bicarbonate, and spot urinary albumin-to-creatinine ratio (uACR). A second blood sample was drawn after 1 h of supine rest, still in fasting conditions, and available in this database for 3,216 patients. Blood samples were collected avoiding fist clenching and transported immediately to the hospital central laboratory. Potassium concentration was measured by indirect potentiometry, with K-selective electrodes on Cobas^®^ analyzer (Roche) simultaneously with hemolysis index. Samples reported as hemolyzed by the laboratory were discarded. The lowest of the two potassium values was used for the analyses in order to minimize imprecision due to pseudohyperkalemia. Hyperkalemia was defined as plasma potassium ≥5.0 mmol/L or the use of potassium binders.

Plasma creatinine was measured with an enzymatic method and standardized to isotope-dilution mass spectrometry. GFR was estimated with the Chronic Kidney Disease-Epidemiology Collaboration (CKD-EPI) equation [20]. GFR was measured using the renal clearance of a radioisotope tracer (either ^51^Cr-EDTA or ^99m^Tc-DTPA), averaging 6–7 consecutive mini-equilibrium 30-min periods, as previously described [21]. In case of irregular voiding, plasma clearance of the tracer was calculated from the late disappearance curve using the slope-intercept method with the Bröchner-Mortensen correction [22].

### Statistical Analysis

Categorical variables are reported as numbers and percentages and continuous variables as means and their standard deviations or medians and interquartile ranges. The functional form of continuous covariates was examined using restricted cubic splines with four degrees of freedom. Nonlinearity was assessed using likelihood ratio tests comparing spline and linear models. When the test for nonlinearity was not statistically significant, the covariate was modeled as a linear term.

Crude and adjusted odds ratios (OR) and their 95% confidence intervals (CI) for the risk of hyperkalemia were estimated with logistic regression models using the following covariates: measured GFR, age, sex, sub-Saharan African ancestry, BMI, history of diabetes, plasma sodium and bicarbonate, uACR, treatment with RAASi, β-blockers, loop and thiazide diuretics, calcineurin inhibitors. Missing values for these variables (<7%) were handled with multiple imputation with chained equations using 5 datasets, under the assumption that data were missing at random.

We tested for interactions between measured GFR, diabetes, and the use of RAASi, as well as between measured GFR and uACR. Sensitivity analyses using an alternative definition of hyperkalemia (plasma potassium ≥4.5 mmol/L), excluding patients with hypokalemia (plasma potassium ≤3.5 mmol/L or those receiving potassium supplements), using only the first plasma potassium measurement, and using estimated instead of measured GFR were performed. In addition, we conducted a subgroup analysis restricted to kidney transplant recipients. Analyses were performed with R software (R Development Core Team, R Foundation for Statistical Computing, Vienna, Austria). A two-sided *p* value of <0.05 was considered statistically significant.

## Results

A total of 5,046 patients were included. Mean age was 52 ± 15 years; 42% were women, 22% were of sub-Saharan African ancestry, 25% had diabetes, and 36% were kidney transplant recipients. Mean measured GFR was 59 ± 26 mL/min/1.73 m^2^, and mean plasma potassium concentration was 4.0 ± 0.5 mmol/L (Table 1). Plasma potassium was higher in males and in advanced CKD (online suppl. Fig. 1, 2; for all online suppl. material, see https://doi.org/10.1159/ajn/ablag008). The overall prevalence of chronic hyperkalemia was 6.4% (95% CI, 5.7–7.1) and increased with the decline of kidney function, ranging from less than 2% in patients with measured GFR >60 mL/min/1.73 m^2^ to 8% in those with GFR between 30 and 60 mL/min/1.73 m^2^ and 26% in those with GFR <20 mL/min/1.73 m^2^ (Fig. 2). Among patients with hyperkalemia, 175 had K^+^ ≥5.0 mmol/L (3.5%), 182 were receiving potassium binders (3.6%), and 36 (0.7%) met both criteria. When alternative definitions of hyperkalemia were used, the prevalence was 4.1% (95% CI, 3.6–4.7) with a threshold of ≥5.5 mmol/L and 3.7% (95% CI, 3.2–4.2) with a threshold of ≥6.0 mmol/L.

**Table 1.** Patient characteristics in the total population and according to the presence of hyperkalemia

Variable	Overall (*n* = 5,046)	No hyperkalemia (*n* = 4,725)	Hyperkalemia (*n* = 321)
Female sex	2,103 (42)	1,999 (42)	104 (32)
Sub-Saharan African ancestry	1,114 (22)	1,065 (23)	49 (15)
Age, years	52±15	52±15	58±13
BMI, kg/m^2^	26.2±5.4	26.2±5.3	26.9±6.4
Systolic blood pressure, mm Hg	130±19	130±19	140±21
Diastolic blood pressure, mm Hg	75±12	75±11	76±12
History of hypertension	3,407 (68)	3,106 (66)	301 (94)
Controlled office blood pressure (<140/90 mm Hg)	3,501 (69)	3,338 (71)	163 (51)
Chronic heart failure	162 (3)	147 (3)	15 (5)
Kidney transplant	1,807 (36)	1,676 (36)	131 (41)
Solitary kidney	231 (5)	212 (5)	19 (6)
Polycystic kidney disease	311 (6)	299 (6)	12 (4)
Diabetes			
No diabetes	3,778 (75)	3,609 (76)	169 (53)
Diabetes without insulin	606 (12)	555 (12)	51 (16)
Diabetes with insulin	662 (13)	561 (12)	101 (32)
HIV	421 (8)	410 (9)	11 (3)
Liver cirrhosis	262 (5)	207 (4)	55 (17)
RAASi	1,925 (38)	1,700 (36)	225 (70)
β-blockers	1,709 (34)	1,545 (33)	164 (51)
Loop diuretics	603 (12)	524 (11)	79 (25)
Thiazide diuretics	384 (8)	346 (7)	38 (12)
Potassium sparing diuretics	55 (1)	49 (1)	6 (2)
SGLT2 inhibitors	55 (1)	49 (1)	6 (2)
Calcineurin inhibitors	1,910 (38)	1,733 (37)	177 (55)
Potassium supplements	223 (4)	223 (5)	0
Any corticosteroid	1,570 (31)	1,476 (31)	94 (29)
NSAIDs	94 (2)	91 (2)	3 (1)
Measured GFR, mL/min/1.73 m^2^	59±26	61±26	34±16
Plasma creatinine, µmol/L	124±64	119±60	185±87
Plasma sodium, mmol/L	140±3	140±3	139±3
Plasma potassium, mmol/L	4.0±0.5	3.9±0.4	4.8±0.7^a^
Plasma bicarbonate, mmol/L	25±3	25±3	23±4
uACR, mg/mmol	2.5 [0.9; 12.1]	2.3 [0.8; 10.0]	12.4 [2.8; 63.8]

Categorical variables are presented as number (percentage) and continuous variables as mean ± SD or median [IQR], as appropriate.

BMI, body mass index; GFR, glomerular filtration rate; NSAIDs, non-steroidal anti-inflammatory drugs; RAASi, renin-angiotensin-aldosterone system inhibitor; SGLT2, sodium glucose cotransporter 2; uACR, spot urinary albumin-to-creatinine ratio.

^a^ The mean plasma potassium level was 5.3 ± 0.3 mmol/L among patients with K^+^ ≥5.0 mmol/L and 4.8 ± 0.7 mmol/L among those receiving potassium binders.

**Fig. 2.**
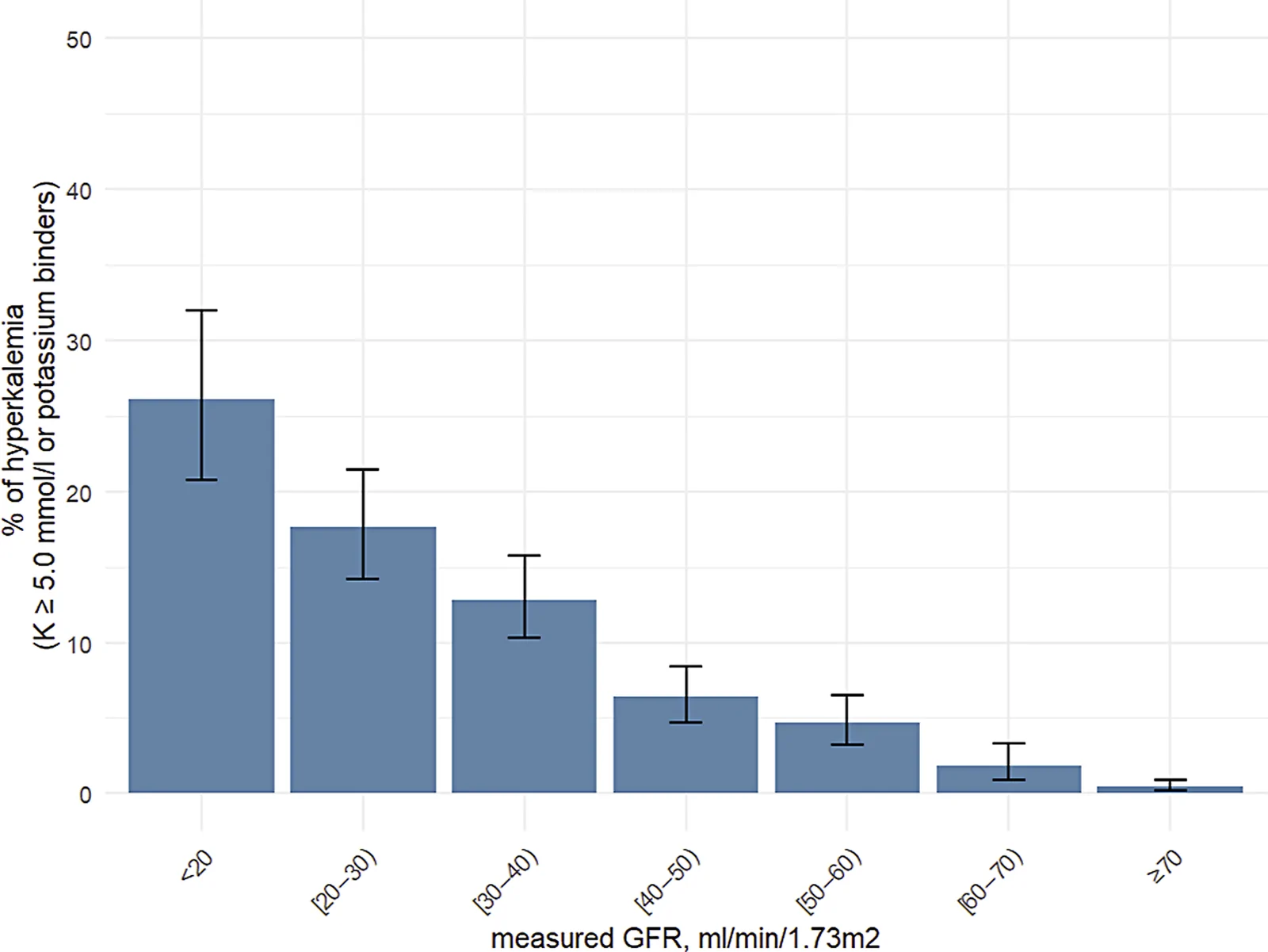
Prevalence of hyperkalemia according to measured GFR.

Patients with hyperkalemia were older, more often male, less frequently of sub-Saharan African ancestry, and more likely to have diabetes, uncontrolled hypertension, lower measured GFR, higher uACR, and lower plasma bicarbonate concentration. They were also more frequently treated with RAASi, β-blockers, calcium channel inhibitors, potassium-wasting diuretics, insulin, and calcineurin inhibitors (Table 1).

In crude analyses (Table 2), hyperkalemia was associated with older age, male sex, higher BMI, diabetes, arterial hypertension, lower measured GFR, lower plasma bicarbonate levels, higher uACR, and the use of RAASi, β-blockers, potassium-wasting diuretics, and calcineurin inhibitors. Conversely, female sex, African origin, and higher plasma sodium levels were protective. After multivariable adjustment, lower measured GFR and higher uACR remained associated with hyperkalemia (OR 1.65 [1.51; 1.81] per 10 mL/min/1.73 m^2^ decrease and 1.07 [1.01; 1.13] per 2-fold increase, respectively). Female sex and sub-Saharan African ancestry were associated with a lower risk of hyperkalemia (OR 0.71 [0.55; 0.92] and 0.68 [0.48; 0.94], respectively). Each 1 mmol/L decrease in plasma bicarbonate was associated with increased risk of hyperkalemia (OR 1.05 [1.01; 1.07]). Diabetes and cirrhosis were also associated with hyperkalemia (OR 1.67 [1.38; 2.17] and 2.32 [1.59; 3.34], respectively). Arterial hypertension was not associated with hyperkalemia after multivariable adjustment (OR 1.42 [0.83; 2.54]). Use of RAASi (OR 2.30 [1.72; 3.11]) and calcineurin inhibitors (OR 1.39 [1.06; 1.83]) was also linked to increased risk, whereas loop diuretics were associated with a reduced risk (OR 0.71 [0.51; 0.98]), as illustrated in Figure 3.

**Table 2.** OR (95% CI) of hyperkalemia associated with patient characteristics in crude and adjusted logistic regression models

​	Crude		Adjusted	
variable	OR [95% CI]	*p* value	OR [95% CI]	*p* value
Age, per 10 year increase	1.35 [1.24; 1.46]	<0.001	1.01 [0.91; 1.11]	0.89
Female sex	0.66 [0.52; 0.83]	<0.001	0.71 [0.55; 0.92]	0.01
Sub-Saharan African ancestry	0.60 [0.44; 0.82]	<0.001	0.68 [0.48; 0.94]	0.02
BMI, per 1 kg/m^2^ increase	1.02 [1.00; 1.04]	0.01	0.98 [0.96; 1.01]	0.07
Kidney transplant	1.24 [0.99; 1.56]	0.06	​	​
ADPKD	0.58 [0.30; 1.00]	0.07	​	​
Diabetes	2.89 [2.30; 3.63]	<0.001	1.67 [1.38; 2.17]	<0.001
Arterial hypertension	7.84 [5.11; 12.79]	<0.001	1.42 [0.83; 2.54]	0.21
Heart failure	1.53 [0.85; 2.54]	0.13	​	​
HIV	0.37 [0.19; 0.65]	0.001	0.60 [0.30; 1.11]	0.13
Cirrhosis	4.51 [3.25; 6.19]	<0.001	2.32 [1.59; 3.34]	<0.001
Measured GFR,per 10 mL/min/1.73 m^2^ decrease	1.78 [1.66; 1.91]	<0.001	1.65 [1.51; 1.81]	<0.001
uACR, per 2-fold increase	1.34 [1.28; 1.40]	<0.001	1.07 [1.01; 1.13]	0.03
Plasma bicarbonate, per 1 mmol/L decrease	1.16 [1.13; 1.18]	<0.001	1.05 [1.01; 1.07]	0.01
Plasma sodium, per 1 mmol/L increase	0.93 [0.90; 0.96]	<0.001	0.98 [0.95; 1.03]	0.54
RAASi	4.04 [3.18; 5.17]	<0.001	2.30 [1.72; 3.11]	<0.001
β-blockers	2.16 [1.72; 2.70]	<0.001	0.93 [0.72; 1.20]	0.54
Loop diuretics	2.60 [1.98; 3.38]	<0.001	0.71 [0.51; 0.98]	0.04
Thiazide diuretics	1.70 [1.18; 2.39]	0.01	0.93 [0.62; 1.37]	0.67
Potassium sparing diuretics	0.76 [0.30; 1.59]	0.51	​	​
Corticosteroids	0.91 [0.71; 1.16]	0.46	​	​
NSAIDs	0.48 [0.12; 1.29]	0.21	​	​
Calcineurin inhibitors	2.10 [1.68; 2.64]	<0.001	1.39 [1.06; 1.83]	0.02

ADPKD, autosomal dominant polycystic kidney disease; BMI, body mass index; GFR, measured glomerular filtration rate; NSAIDs, non-steroidal anti-inflammatory drugs; RAASi, renin-angiotensin-aldosterone system inhibitor; uACR, urinary albumin-to-creatinine ratio.

**Fig. 3.**
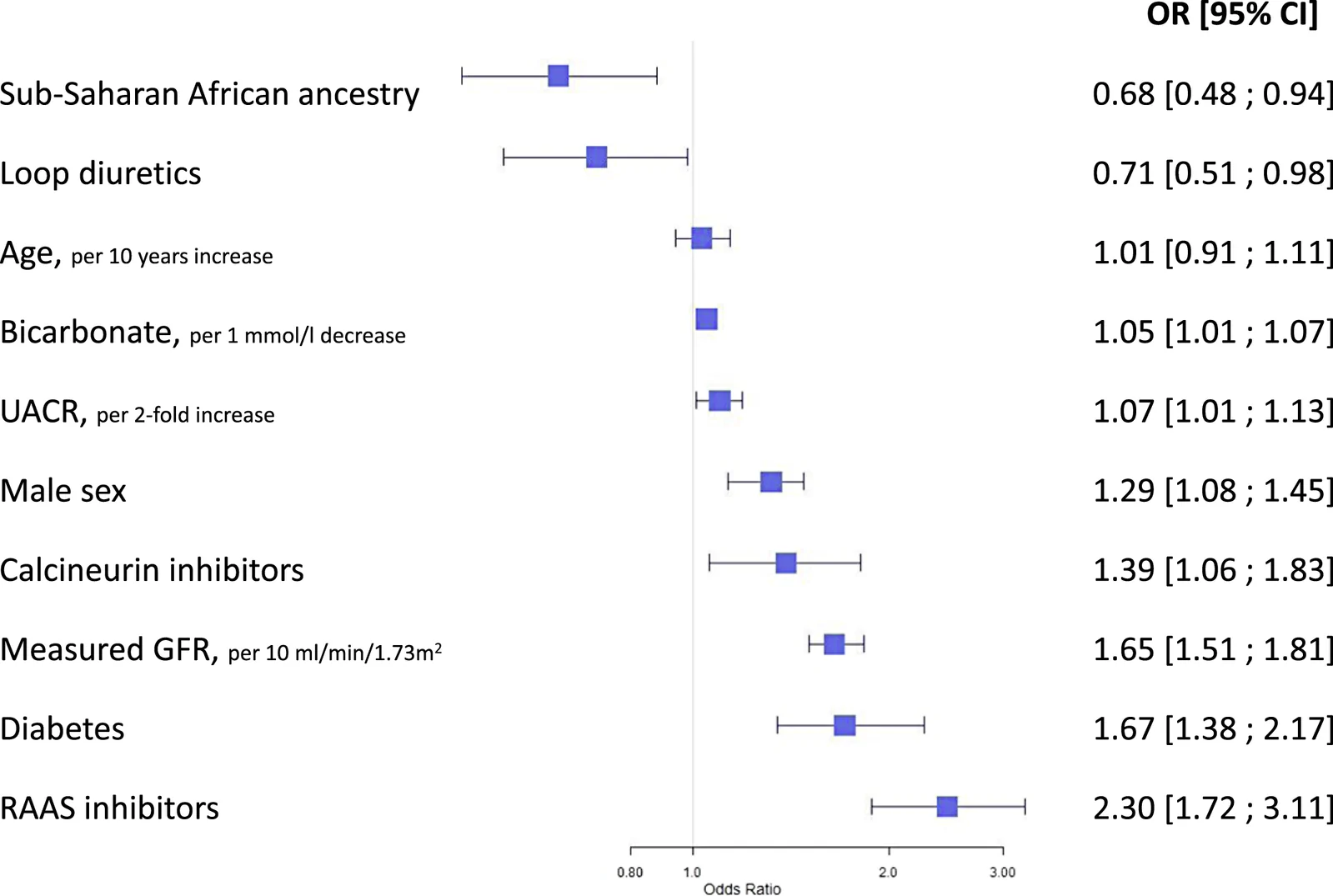
Multivariable OR for hyperkalemia in patients with non-dialysis CKD.

In spline-based logistic regression analyses, the association between measured GFR and hyperkalemia was linear. The adjusted odds of hyperkalemia increased progressively with declining measured GFR, with a steeper rise observed below approximately 30 mL/min/1.73 m^2^, without evidence of a sharp threshold effect (online suppl. Fig. 3).

We observed a significant interaction between measured GFR and RAASi use (*p* = 0.036). The effect of lower GFR on the risk of hyperkalemia was stronger in RAASi users (OR 1.82 [1.61–2.07] per 10 mL/min/1.73 m^2^ decrease in measured GFR) than in non-users (OR 1.43 [1.25–1.66]). There were no significant interactions between GFR and diabetes (*p* = 0.14) or diabetes and RAASi (*p* = 0.93), or GFR and uACR (*p* = 0.67).

Sensitivity analyses using a different definition of hyperkalemia (plasma potassium ≥4.5 mmol/L or prescription of potassium binders) or excluding patients on potassium supplements and plasma potassium ≤3.5 mmol/L, as well as using only the first plasma potassium measurement produced virtually unchanged results (online suppl. Tables–3). Sensitivity analyses using estimated rather than measured GFR yielded overall qualitatively similar findings, but with attenuated effect estimates for kidney function (OR 1.40 vs. 1.65 per 10 mL/min/1.73 m^2^ decrease) and for the protective role of female sex (OR 0.80 vs. 0.71), whereas the association with albuminuria was more pronounced when using estimated rather than measured GFR (OR 1.13 vs. 1.07 per twofold increase in uACR) (online suppl. Table 4).

Subgroup analysis in kidney transplant patients demonstrated that lower kidney function was the strongest factor associated with hyperkalemia (OR 1.73, 95% CI 1.48–2.02; *p* < 0.001), consistent with the main analysis (online suppl. Table 5). Diabetes and RAASi use were also independently associated with increased odds of hyperkalemia, whereas female sex, sub-Saharan African ancestry, and loop diuretics were associated with lower odds.

## Discussion

In this large cross-sectional study of non-dialysis CKD patients who underwent GFR measurement and simultaneous, fasting, standardized plasma potassium measurement, we described the prevalence of chronic outpatient hyperkalemia and its associated factors. After adjustment for confounders, lower measured GFR, higher albuminuria, lower plasma bicarbonate concentration, diabetes, the use of RAASi and calcineurin inhibitor were independently associated with a higher prevalence of hyperkalemia, whereas female sex, sub-Saharan African ancestry, and the use of loop diuretics were associated with a lower prevalence. Neither age nor BMI were independently associated with the prevalence of hyperkalemia after adjustment for measured GFR.

The prevalence of hyperkalemia in our study was relatively low (6.4%) and increased with CKD severity, consistent with the well-established decline in potassium excretion as kidney function worsens [23]. Our estimates fall toward the lower end of the broad range reported in the literature, where the prevalence of hyperkalemia varies substantially across CKD populations, clinical settings, and thresholds used to define hyperkalemia, from less than 10% to more than 50% [1, 13, 18, 24, 25]. Unlike most previous studies, potassium was measured under strictly standardized conditions: in fasting patients at rest, using plasma samples (typically lower than serum concentrations [26, 27]), analyzed locally, without delay, with repeat testing after 1 h to minimize pseudohyperkalemia [28, 29]. Our study population was derived from systematically characterized clinical visits with simultaneous measurements, in contrast to many real-world datasets that rely on opportunistic laboratory values or administrative coding [16, 30]. In addition to dosage-related factors, differences in comorbidity profiles, medication exposure, and dietary practices across cohorts likely contribute to heterogeneity in reported prevalence. Many prior studies included patients with acute illness, hospitalization, or advanced cardiorenal syndromes, while our cohort predominantly comprised stable, ambulatory patients.

The use of measured rather than estimated GFR distinguishes our study from most previous investigations of hyperkalemia in CKD. Although directly measured GFR is not routinely available in everyday clinical practice, its use in this study provides a unique opportunity to more accurately assess the independent contributions of kidney function and demographic characteristics to hyperkalemia risk. In our cohort, measured GFR was a strong independent predictor of hyperkalemia, with a 65% higher prevalence per 10 mL/min/1.73 m^2^ decrease. Of note, the effect of GFR was slightly attenuated when using estimated GFR instead of measured GFR. Previous studies have reported comparable effect sizes; for example, a meta-analysis of more than 1.2 million participants from 27 international cohorts by Kovesdy et al. [1] showed that a 15 mL/min decrease in eGFR approximately doubled the odds of hyperkalemia. The independent contribution of albuminuria to the risk of hyperkalemia remains a debated issue. In the abovementioned meta-analysis [1], the association between uACR and hyperkalemia was most apparent in non-CKD cohorts; in CKD cohorts, lower eGFR strongly predicted potassium >5.5 mmol/L whereas higher uACR showed only weak or no association. In contrast, in the post hoc analyses from the FIDELIO-DKD Trial, increased uACR was a risk factor for hyperkalemia independently of GFR level, while other studies reported that only severe albuminuria significantly increases the risk of hyperkalemia [4, 5]. In our study, albuminuria remained associated with hyperkalemia after adjustment for measured GFR and other covariates, with no evidence of interaction between measured GFR and uACR; however, its effect size was modest compared with that of measured GFR, suggesting that potassium disturbances are primarily driven by reduced kidney function.

The association between age, sex, and hyperkalemia is of major clinical relevance but remains debated. In unadjusted or minimally adjusted analyses, older age is frequently identified as a risk factor for hyperkalemia [31, 32]. However, other studies suggest that age does not independently predict hyperkalemia after adjustment for GFR and other confounders [1, 33, 34]. Female sex was found to be protective in most [1, 3, 35] but not all analyses [14, 18, 32, 36]. Remarkably, analyses from the large FIDELIO-DKD trial reported opposite patterns, with older age and male sex both associated with a lower risk of hyperkalemia, with no clear explanation for this finding [17]. One potential explanation for inconsistent findings across CKD cohorts may be that these variables are components of GFR estimating equations, which may bias analyses attempting to evaluate their effects independently of kidney function, especially for age. In our study, age was not independently associated with hyperkalemia once measured GFR was taken into account, while female sex remained strongly and independently associated with a lower risk. Our findings support the hypothesis that the observed relationship between older age and hyperkalemia in CKD is largely mediated by age-related decline in kidney function and the accumulation of comorbidities and medication exposure, rather than age itself. When kidney function is accurately quantified and major clinical confounders are accounted for, chronological age per se contributes little additional information to hyperkalemia risk. In contrast, we confirm that female sex is associated with reduced risk of hyperkalemia. This association was more pronounced when using measured rather than estimated GFR, illustrating how creatinine-based GFR estimating equations may impact the analysis of the independent contribution of demographic characteristics. Underlying proposed mechanisms include greater aldosterone responsiveness [37], more efficient distal tubular potassium secretion [38], and lower intracellular potassium stores related to lower muscle mass. Differences in dietary potassium intake between men and women could also contribute; however, this hypothesis requires dedicated investigation with assessment of potassium intake to clarify the relative contributions of intake versus kidney handling.

Consistent with previous studies, diabetes [2, 16, 35] and metabolic acidosis [14] were independently associated with a higher prevalence of hyperkalemia, whereas sub-Saharan African ancestry was associated with a lower prevalence [13, 34, 39]. BMI was not independently associated with hyperkalemia in our study, in line with previous CKD cohorts [1, 23]. However, some studies have reported an increased risk of hyperkalemia among individuals with low BMI [13, 40], possibly reflecting underlying frailty or reduced drug distribution volume. However, the impact of BMI should be interpreted with great caution when GFR is estimated from creatinine-derived equation and even when it is measured using plasma clearance. The AASK study [13] (where GFR was actually measured by ^125^I-iothalamate clearance) found low BMI to be a risk factor for hyperkalemia, but the authors interpreted that GFR might be overestimated at low BMI.

In the present study, the use of RAASi increased the risk of hyperkalemia more than 2-fold. This finding aligns with extensive observational and trial-based evidence showing that RAAS blockade is one of the strongest predictors of hyperkalemia in CKD [41]. Importantly, however, permanently discontinuing RAASi in response to hyperkalemia is counterproductive, leading to higher rates of cardiovascular events and accelerated kidney function decline [15, 42]. Notably, loop diuretics were associated with a lower risk of hyperkalemia in our cohort. The reverse association in unadjusted analyses is consistent with expected confounding by indication as loop diuretics are more frequently prescribed to patients with more advanced CKD or higher baseline potassium levels. Taken together, these findings underscore the well-recognized therapeutic trade-off: RAASis provide substantial cardiorenal protection but at the cost of significantly increased kalemia, underscoring the need for close monitoring and pharmacological strategies to avoid premature discontinuation of RAAS blockade.

We found similar results using a plasma potassium threshold of 4.5 mmol/L (the upper limit of our laboratory). Current guidelines do not provide a universal biochemical definition of hyperkalemia: the 2024 KDIGO guideline [43] emphasizes clinical context rather than a specific numeric threshold, whereas the ESC [44, 45] generally considers serum potassium levels >5.0 mmol/L as hyperkalemia. Importantly, these recommendations are largely based on serum – and not plasma – measurements which yield on average higher values [27]. Overall, analyses from large cohorts show that the lowest risk is observed at plasma potassium levels of 4.0–5.0 mmol/L with a progressive increase in risk below and above this range [1, 46].

Several limitations of the present study should be acknowledged. First, its cross-sectional design precludes causal inference and, in the absence of longitudinal follow-up, does not allow assessment of prognostic implications or temporal relationships, prognostic implications, or long-term clinical outcomes, including recurrent hyperkalemia, hospitalization, RAAS inhibitor discontinuation, cardiovascular events, or mortality. Second, the limited use of SGLT2 inhibitors during the study period may limit the generalizability of our findings to contemporary practice. Likewise, there was a very low prescription rate of mineralocorticoid receptor antagonists in our study. In addition to the low prevalence of heart failure, they were only rarely prescribed as antihypertensive therapy, further contributing to their low overall use. This likely reflects real-world prescribing patterns as aldosterone antagonists remain underused in patients with resistant hypertension despite guideline recommendations, even among those with an eGFR >45 mL/min/1.73 m^2^ [47, 48]. Consistent with this, we previously reported in the closely related hospital-based NephroTest cohort that only 2.8% of hypertensive patients received aldosterone antagonists, although their use was independently associated with better blood pressure control in this population [49]. Therefore, the limited use of mineralocorticoid receptor antagonists in our cohort is unlikely to reflect a selection bias specific to the present study but rather contemporary prescribing practices. Third, information on dietary potassium intake was not available. Dedicated studies incorporating dietary assessment – either through questionnaires or 24-h urinary potassium excretion – are needed to better delineate the impact of potassium intake on kalemia. Fourth, our findings relate primarily to sustained hyperkalemia identified during routine outpatient assessment and may not be generalizable to acute hyperkalemic episodes, which have distinct precipitating factors and may carry a different risk of morbidity and mortality. Fifth, the absence of information on treatment indications precludes exclusion of confounding by indication. Because these agents are commonly prescribed in response to hyperkalemia, their true protective effect may have been underestimated. Finally, our population was drawn from a nephrology referral cohort, which may restrict applicability to broader CKD populations with different comorbidity profiles or treatment patterns. Patients with hyperkalemia or a perceived higher risk of hyperkalemia may have been more likely to be referred for specialized evaluation, potentially limiting the generalizability of the prevalence estimates and the distribution of associated risk factors, but unlikely for association estimates themselves.

The major strength of this study is the use of measured rather than estimated GFR in a large, exceptionally well-phenotyped CKD cohort, allowing a more robust assessment of independent risk factors than studies relying on creatinine-based estimating equations. Combined with standardized blood and urine analyses and detailed clinical, biochemical, and treatment data, this approach enabled robust multivariable modelling. Furthermore, the systematic nature of data collection minimized missing data and information bias, strengthening confidence in the observed associations.

Our findings have important clinical implications. They suggest that outpatient hyperkalemia surveillance should primarily be guided by kidney function together with diabetes, albuminuria, metabolic acidosis, and RAAS inhibitor use, rather than demographic characteristics such as age or BMI. Accurate assessment of kidney function appears particularly important for individualized risk stratification, especially when decisions regarding RAAS inhibitor continuation, potassium monitoring, or preventive interventions are considered.

## Conclusions

This large cross-sectional study of CKD patients with measured GFR provides a comprehensive description of the prevalence of hyperkalemia and its main determinants. After adjustment for confounders, lower measured GFR, metabolic acidosis, diabetes, and RAASi use emerged as the strongest independent predictors of hyperkalemia. Conversely, female sex, sub-Saharan African ancestry, and loop-diuretic use were associated with a markedly lower risk. Overall, these findings refine our understanding of hyperkalemia risk in CKD and highlight the importance of accurately assessing kidney function when investigating independent determinants of hyperkalemia. Further longitudinal studies, including potassium intake evaluation, are needed to clarify causal pathways and to determine how these factors influence clinical outcomes over time.

## Statement of Ethics

The study was approved by the local Ethics Committee (Comité d’Evaluation de l’Ethique des projets de Recherche Biomédicale Paris Nord, Institutional Review Board [IRB 00006477], Assistance Publique-Hôpitaux de Paris). All patients signed informed consent for the scientific use of anonymized data. The study was conducted in accordance with good clinical practices and the Declaration of Helsinki.

## Conflict of Interest Statement

The authors have no conflicts of interest to declare.

## Funding Sources

This study was not supported by any sponsor or funder.

## Author Contributions

All authors contributed to the study conception and design. J.M., M.F., and E.V.-P. had full access to all of the data in the study and performed statistical analyses and designed tables and figures. J.M., M.F., A.L., T.F., and E.V.-P. collected the data. J.M., B.S., N.A.P., and E.V.-P. designed the study, interpreted the data, performed statistical analyses, performed the literature search, and wrote the manuscript. A.L., T.F., F.V., B.S., E.V.-P., N.T., A.B., and N.A.P. contributed substantially to data collection and interpretation. B.S. and N.A.P. contributed to data interpretation and statistical analysis. All authors reviewed and provided critical comments on the manuscript and gave final approval for submission of the manuscript.

## Data Availability

The data that support the findings of this study are not publicly available due to privacy reasons but are available from the corresponding author upon request.
